# Author Correction: The molecular mechanism of snake short-chain α-neurotoxin binding to muscle-type nicotinic acetylcholine receptors

**DOI:** 10.1038/s41467-024-48904-y

**Published:** 2024-05-28

**Authors:** Mieke Nys, Eleftherios Zarkadas, Marijke Brams, Aujan Mehregan, Kumiko Kambara, Jeroen Kool, Nicholas R. Casewell, Daniel Bertrand, John E. Baenziger, Hugues Nury, Chris Ulens

**Affiliations:** 1 Laboratory of Structural Neurobiology, Department of Cellular and Molecular Medicine, Faculty of Medicine, KU Leuven, 3000 Leuven, Belgium; 2 University Grenoble Alpes, CNRS, CEA, IBS, F-38000 Grenoble, France; 3 University Grenoble Alpes, CNRS, CEA, EMBL, ISBG, F-38000 Grenoble, France; 4 HiQscreen, 1222 Vésenaz, Geneva, Switzerland; 5 AIMMS Division of BioMolecular Analysis, Vrije Universiteit Amsterdam, 1081 HV Amsterdam, Netherlands; 6 Centre for Snakebite Research & Interventions, Liverpool School of Tropical Medicine, L3 5QA Liverpool, UK; 7 Department of Biochemistry, Microbiology, and Immunology, University of Ottawa, Ottawa, ON K1H 8M5 Canada

**Keywords:** Cryoelectron microscopy, Ligand-gated ion channels

Correction to: *Nature Communications* 10.1038/s41467-022-32174-7, published online 04 August 2022

In the original version of this Article, the Acknowledgements section incorrectly omitted two of the funding sources. A sentence ‘CU was supported by grants G0C1319N and G087921N from FWO-Vlaanderen.’ has been now added. This has been corrected in both the PDF and HTML versions of the Article.

