# Supplementary material for: The molecular mechanism of snake short-chain α-neurotoxin binding to muscle-type nicotinic acetylcholine receptors
Source: Nat Commun. 2022 Aug 4;13:4543. doi: 10.1038/s41467-022-32174-7 (PMC9352773; doi:10.1038/s41467-022-32174-7)
Supplement: Supplementary file 1 — Supplementary Information [file 41467_2022_32174_MOESM1_ESM.pdf]

Supplementary Information

The molecular mechanism of snake short-chain α-neurotoxin binding to muscle-type nicotinic acetylcholine receptors

Mieke Nys[1*#], Eleftherios Zarkadas[2,3#], Marijke Brams[1], Aujan Mehregan[1], Kumiko Kambara[4], Jeroen Kool[5], Nicholas R. Casewell[6], Daniel Bertrand[4], John E. Baenziger[7], Hugues Nury[2], Chris Ulens[1*]

[1] Laboratory of Structural Neurobiology, Department of Cellular and Molecular Medicine, Faculty of Medicine, KU Leuven, 3000 Leuven, Belgium

[2] Univ. Grenoble Alpes, CNRS, CEA, IBS, F-38000 Grenoble

[3] Univ. Grenoble Alpes, CNRS, CEA, EMBL, ISBG, F-38000 Grenoble

[4] HiQscreen, 1222 Vésenaz, Geneva, Switzerland

[5] AIMMS Division of BioMolecular Analysis, Vrije Universiteit Amsterdam, 1081 HV Amsterdam, Netherlands

[6] Centre for Snakebite Research & Interventions, Liverpool School of Tropical Medicine, L3 5QA Liverpool, United Kingdom

[7] Department of Biochemistry, Microbiology, and Immunology, University of Ottawa, Ottawa, ON, K1H 8M5, Canada

* Correspondence and requests for materials should be addressed to M.N. (mieke.nys@kuleuven.be) or to C.U. (chris.ulens@kuleuven.be).

[#] These authors contributed equally to this work.

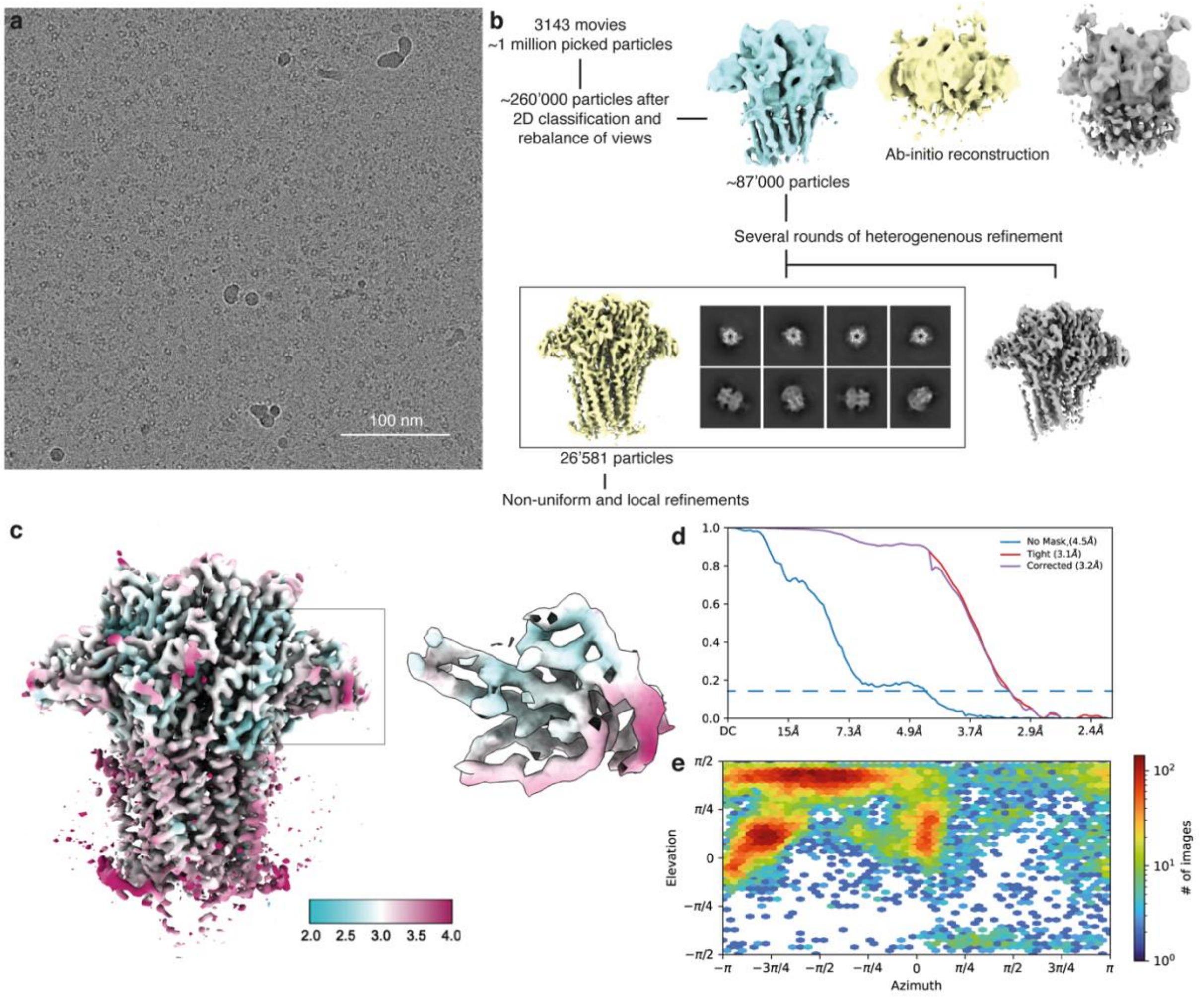

Supplementary Figure 1. Cryo-EM processing and map-model parameters. (**a**) Example micrograph (out of 3143 motion-corrected micrographs) of the imaged *Torpedo* nAChR in complex with ScNtx. (**b**) Schematic of the image analysis workflow, including 2D class averages. (**c**) Side view of the sharpened final reconstruction, colored according to the local resolution. (**d**) Gold-standard Fourier shell correlation (FSC) curves. The dotted line represents the 0.143 FSC threshold. (**e**) Heat map of the angular distribution of particle projections for the *Torpedo* nAChR-ScNtx reconstruction.

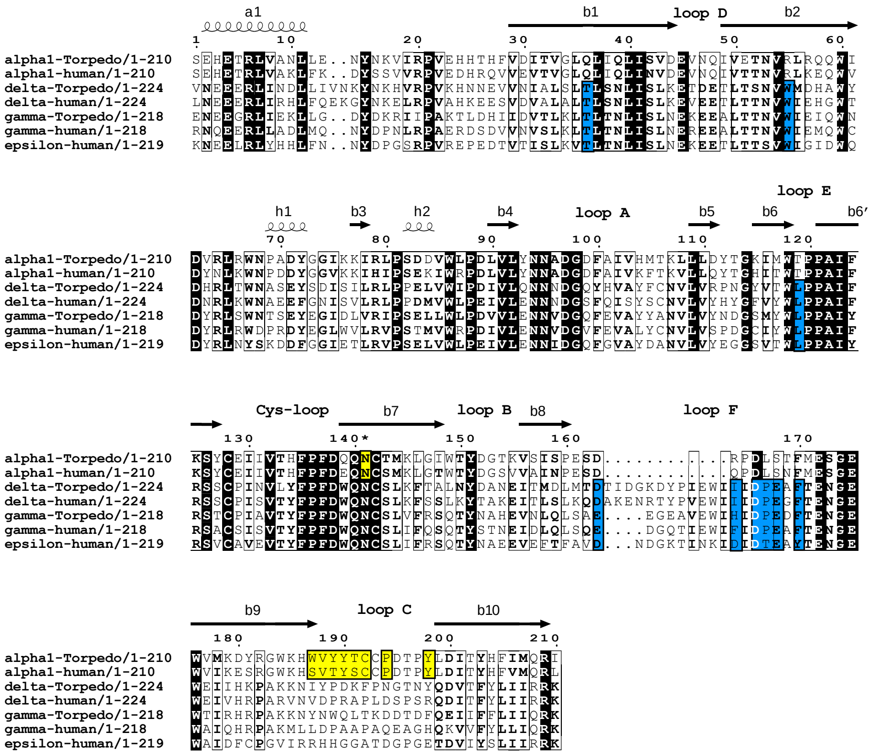

Supplementary Figure 2. Multiple sequence alignment of human and *Torpedo* muscle-type nAChR subunits. Residue numbering and secondary structure information at the top is for the *Torpedo* α1 subunit. Black boxes indicate strict conservation. Characters in bold indicate similarity. Frames indicate regions of similarity. Interacting residues from the principal subunit are highlighted in yellow, interacting residues from the complementary subunit in blue. *Glycosylation site

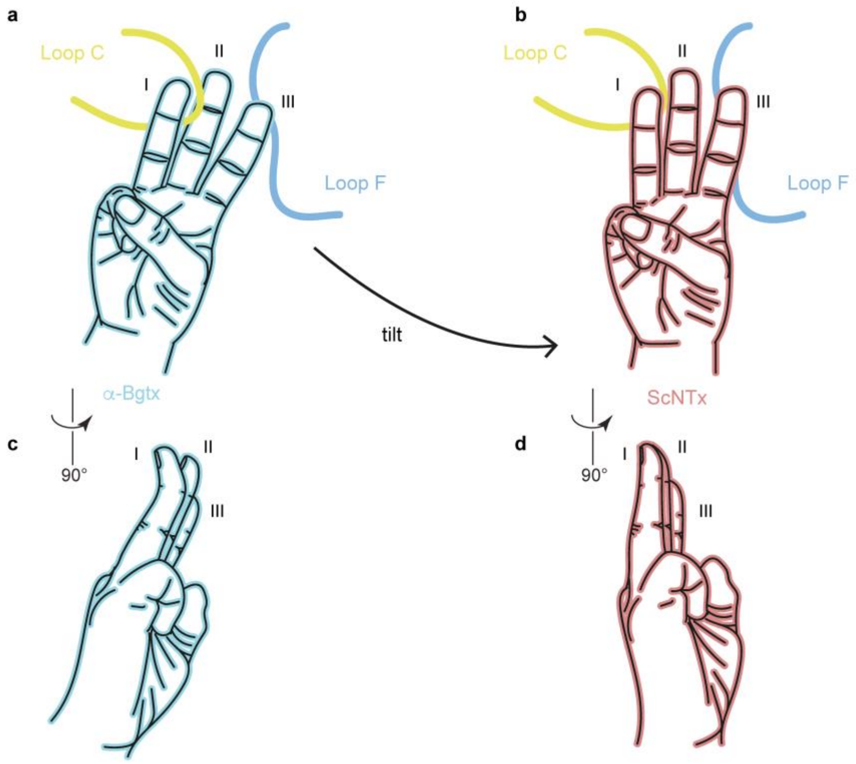

Supplementary Figure 3. Comparison of the ScNtx and α-Bgtx binding mode to the *Torpedo* nAChR: simplified view. α-Bgtx (cyan) and ScNtx (salmon) are depicted as three-fingered hands. Roman numbers indicate finger I-III. (**a** and **b**) ScNtx and α-Bgtx are located between loop C from the principal subunit (in yellow) and loop F from the complementary subunit (in blue). (**c** and **d**) α-Bgtx and ScNtx depicted as in (**a** and **b**) but rotated by 90 ° around the z-axis.

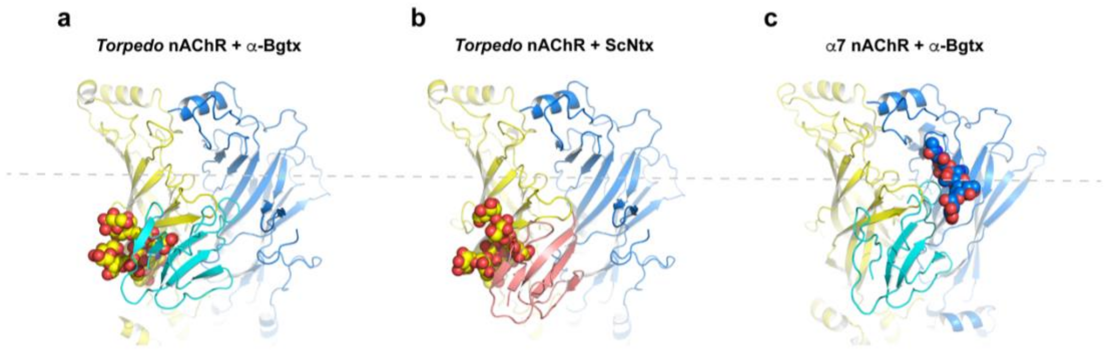

Supplementary Figure 4. The role of N-linked glycans in the binding of α-neurotoxins to nAChRs. Toxins and receptors are shown as cartoons, glycans are depicted as spheres. The dashed line indicates the position of the tip of finger II of α-Bgtx bound to the α7 nAChR. The principal subunit is indicated in yellow, the complementary subunit in blue. α-Bgtx is colored in cyan, ScNtx in salmon. (**a**) α-Bgtx bound to the *Torpedo* nAChR (PDB ID: 6UWZ[3]). (**b**) ScNtx bound to the *Torpedo* nAChR. (**c**) α-Bgtx bound to the neuronal α7 nAChR (PDB ID: 7KOO[4]).

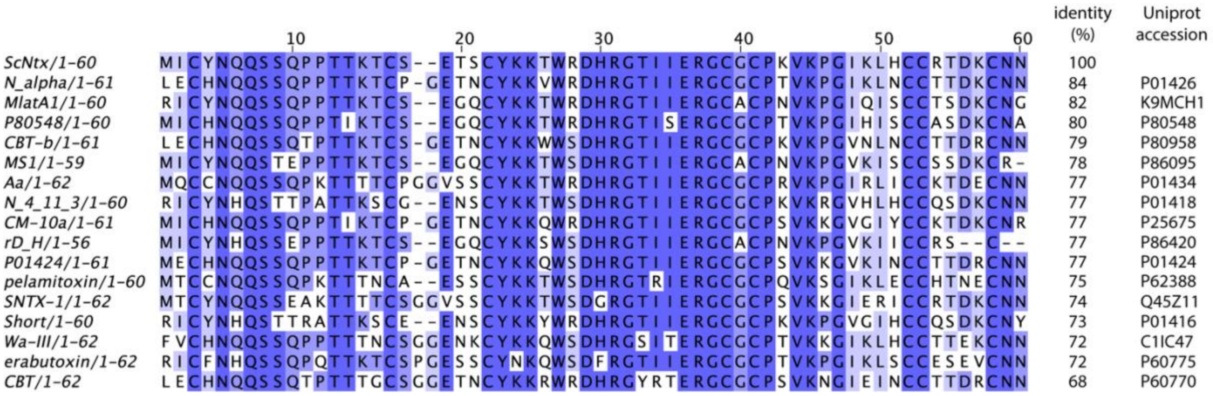

Supplementary Figure 5. Multiple sequence alignment of ScNtx with short-chain α-neurotoxins. Adjusted from [1,2]. Protein sequences were aligned using ClustalO in Jalview. The degree of sequence identity is displayed in shades of blue. Residue numbering at the top is for ScNtx. ScNtx: consensus short-chain α-neurotoxin; N_alpha: Short neurotoxin 1 from *Naja pallida*; MlatA1: Three-finger toxin A1 from *Micrurus laticollaris*; P80548: Three-finger toxin Mnn I from *Micrurus nigrocinctus*, CBT-b: Cobrotoxin-b from *Naja atra*, MS1: Three-finger toxin MS1 from *Micrurus surinamensis*; Aa: Short neurotoxin 1 from *Acanthophis antarcticus*; N_4_11_3: Short neurotoxin 1 from *Dendroaspis viridis*; CM-10a: Short neurotoxin 2 from *Naja haje haje*; rD_H: Frontoxin I from *Micrurus frontalis*; P01424: Short neurotoxin 1 from *Naja melanoleuca*; pelamitoxin: Pelamitoxin a from *Hydrophis platurus*; SNTX-1: Short neurotoxin 1 from *Oxyuranus scutellatus scutellatus*; Short: Short neurotoxin 1 from *Dendroaspis polylepis polylepis*; Wa-III: Three-finger toxin W-III from *Walterinnesia aegyptia*; Erabutoxin: Erabutoxin a from *Laticauda semifasciata*, CBT: Cobrotoxin from *Naja atra*.

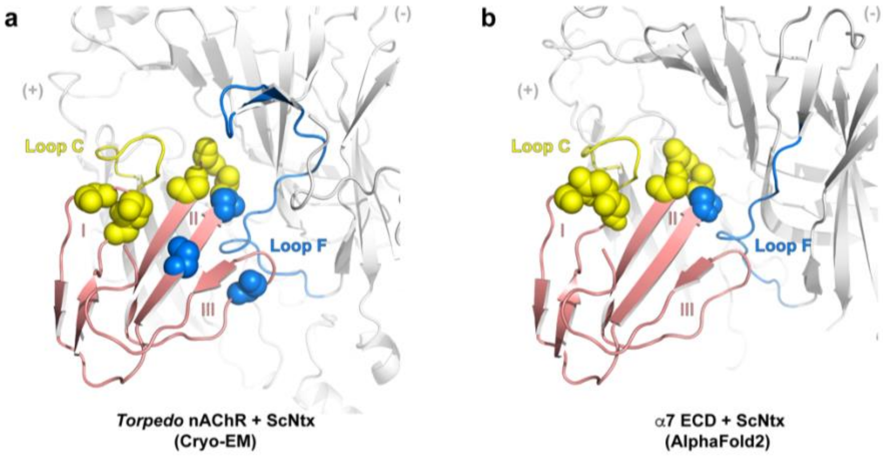

Supplementary Figure 6. Comparison of the toxin-receptor interface of the *Torpedo* nAChR-ScNtx and the α7 nAChR-ScNtx complexes. Toxins and receptors are shown as cartoons. Residues forming H-bonds or salt bridges with loop C or loop F are shown as yellow or blue spheres, respectively. (+) indicates the principal subunit, (-) indicates the complementary subunit. (**a**) Cryo-EM structure of the *Torpedo* nAChR in complex with ScNtx. (**b**) AlphaFold2[22] model of two α7 nAChR ECDs in complex with ScNtx.

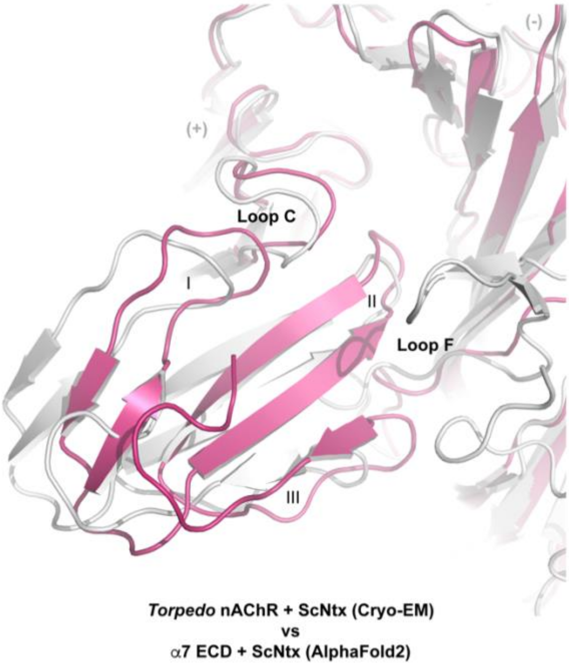

Supplementary Figure 7. Comparison of the binding mode of ScNtx to the muscle-type *Torpedo* nAChR and the neuronal α7 nAChR. Superposition of the Cryo-EM structure of the *Torpedo* nAChR – ScNtx complex (grey) and the AlphaFold2[22] model of two α7 nAChR ECDs in complex with ScNtx (pink). (+) and (-) indicate the principal and complementary subunit, respectively.

Supplementary Table 1. Data collection and refinement statistics.

**Data collection**	
Microscope	Glacios
Magnification	36000
Voltage (ke^-V)	200
Exposure time (s)	4
Number of frames	40
Electron exposure ($e^-/Å^2$)	38
Defocus range (μm)	0.7 to -2.2
Detector	Gatan K2 Summit
Pixel size (Å/pixel)	1.145
Movies	3143
Symmetry imposed	C1
**Reconstruction**	
Initial number of particles	1015654
Final number of particles for reconstruction	26581
Map Resolution (Å)	3.15
FSC threshold	0.143
**Refinement**	
Number of non-H atoms	15865
Protein residues	1914
N-glycan	28
Molprobity score	1.83
Clashscore	9.99
Poor rotamers (%)	0
R.M.S.D. values	
Bond lengths (Å)	0.004
Bond angles (°)	0.656
Ramachandran analysis	
Favored (%)	95.59%
Outliers (%)	0

Supplementary Table 2. Interacting surface area calculations. Calculation of the interacting surfaces for ScNtx and α-Bgtx with the α_{δ} (+) and δ (-) subunit of the *Torpedo* nAChR, expressed in $Å^2$ and % of total interacting surface.

$Å^2$ (% of total IA surface)	**ScNtx**	**α-Bgtx**
**(+)**		
Total	585.75 (56 %)	734.90 (65 %)
Protein	460.75 (44 %)	630.15 (56 %)
Loop C	347.60 (33 %)	480.40 (42 %)
Sugars	142.40 (14 %)	172.40 (15 %)
**(-)**		
Total	456.50 (44 %)	395.80 (35 %)
Loop F	352.50 (34 %)	294.50 (26 %)

Supplementary Table 3. Contacts between ScNtx/ α-Bgtx and the α_γ–γ interface of the *Torpedo* nAChR.

	α-Bgtx	ScNtx	finger #
principal side (α)			
W187	**T6**, A7, T8, S9	**Q7**	I
Y189	T6, **T8**, S9, P10, I11	Q7, S8, Q10	I
	V39, **V40**		II
Y190		**S8**	I
	**H68**		Cterm
	**D30**, F32, R36, G37, K38, V39	**D29**, **R31**, T33, I34	II
T191		**S8**	I
	H68, P69, **K70**		Cterm
	R36, G37, **K38** (x3)	**T33**	II
C192	**R36**		II
P194	I11	S9, **Q10**	I
	H68		Cterm
Y198	R36	R31	II
N-glycan	**T6, A7**		I
	**E41**	**E36**	II
complementary side (γ)			
K34	S34		II
T36	A31		II
W55	A31, F32	H30	II
Y117	S35		II
L119		H30	II
E163	S34	**R28**	II
H172	**D30**, A31	R28, D29	II
D174	D30	D29	II
P175		W27, R28	II
	Y54	K45, P46, G47, I48	III
E176	**W28**	W27	II
		**K45**, I48	III
F178		**K45**	III

H-bonds and salt bridges are shown in bold, Van der Waals interactions are in normal font.
