# Supplementary material for: The molecular mechanism of snake short-chain α-neurotoxin binding to muscle-type nicotinic acetylcholine receptors
Source: Nat Commun. 2022 Aug 4;13:4543. doi: 10.1038/s41467-022-32174-7 (PMC9352773; doi:10.1038/s41467-022-32174-7)
Supplement: Supplementary file 3 — Description of Additional Supplementary Files [file 41467_2022_32174_MOESM3_ESM.pdf]

Description of Additional Supplementary Files

File Name: Supplementary Data 1

Description: Model of the neuronal α7 nAChR in complex with ScNtx predicted by AlphaFold2 using the cryo-EM structure of the α7 nAChR in complex with α-Bgtx as custom template (PDB:7KOO) and based on α7/α7 ECD interface sequences and the ScNtx sequence.

File Name: Supplementary Movie 1

Description: Overall view of the *Torpedo* nAChR—ScNtx complex, receptor-toxin interface and molecular contacts. This movie shows an overall view of the *Torpedo* nAChR—ScNtx complex and zooms in on the toxin-receptor interface across the entire ligandbinding site (αδ−δ). Interacting residues and glycans are shown as sticks, colored by subunit. Dashed lines indicate hydrogen bonds or salt bridges. The α-subunits are colored in yellow, the β-subunit in red, the γ-subunit in cyan, the δ-subunit in blue and ScNtx in salmon.
