# Supplementary material for: The molecular mechanism of snake short-chain α-neurotoxin binding to muscle-type nicotinic acetylcholine receptors
Source: Nat Commun. 2022 Aug 4;13:4543. doi: 10.1038/s41467-022-32174-7 (PMC9352773; doi:10.1038/s41467-022-32174-7)
Supplement: Supplementary file 6 — Reporting Summary [file 41467_2022_32174_MOESM6_ESM.pdf]

nature portfolio

Corresponding author(s): Chris Ulens and Mieke Nys

Last updated by author(s): Jul 4, 2022

Reporting Summary

Nature Portfolio wishes to improve the reproducibility of the work that we publish. This form provides structure for consistency and transparency in reporting. For further information on Nature Portfolio policies, see our Editorial Policies and the Editorial Policy Checklist.

Statistics

For all statistical analyses, confirm that the following items are present in the figure legend, table legend, main text, or Methods section.

n/a	Confirmed	
☐	☒	The exact sample size (*n*) for each experimental group/condition, given as a discrete number and unit of measurement
☐	☒	A statement on whether measurements were taken from distinct samples or whether the same sample was measured repeatedly
☐	☒	The statistical test(s) used AND whether they are one- or two-sided<br>*Only common tests should be described solely by name; describe more complex techniques in the Methods section.*
☒	☐	A description of all covariates tested
☒	☐	A description of any assumptions or corrections, such as tests of normality and adjustment for multiple comparisons
☐	☒	A full description of the statistical parameters including central tendency (e.g. means) or other basic estimates (e.g. regression coefficient) AND variation (e.g. standard deviation) or associated estimates of uncertainty (e.g. confidence intervals)
☐	☒	For null hypothesis testing, the test statistic (e.g. F, t, r) with confidence intervals, effect sizes, degrees of freedom and P value noted<br>*Give P values as exact values whenever suitable.*
☒	☐	For Bayesian analysis, information on the choice of priors and Markov chain Monte Carlo settings
☒	☐	For hierarchical and complex designs, identification of the appropriate level for tests and full reporting of outcomes
☒	☐	Estimates of effect sizes (e.g. Cohen's *d*, Pearson's *r*), indicating how they were calculated

Our web collection on statistics for biologists contains articles on many of the points above.

Software and code

Policy information about availability of computer code

Data collection

1. Nanotemper software: MO.Control v1.6
2. Nanotemper software: MO.Screening Control v1.10
3. Cryo-EM data collection: FEI EPU 1.9 , SerialEM 3.6
4. HiClamp electrophysiology data were acquired with proprietary software written in Matlab from Mathworks Version 2013.

Data analysis

1. Nanotemper software: MO.Affinity Analysis v2.3
2. Cryo-EM data analysis: cryoSPARC v2, crYOLO 1.7.6, Coot 0.9.6, phenix 1.19.2-4158, ccp4 7.1.016, Molprobity 4.5.1, PyMOL Molecular Graphics System 2.5.2, UCSF Chimera 1.8.1, UCSF ChimeraX 1.2.5
3. HiClamp electrophysiology data were analyzed with proprietary software written in Matlab from Mathworks Version 2013.
4. Protein sequences were aligned using ClustalO (version 1.2.4) in Jalview (version 2.10.5) or ESPript 3.0
5. Figures were prepared in Adobe Illustrator 23.1.1 and Adobe Illustrator 26.0
6. Morph movies were prepared in PyMOL 2.5.2 and Quicktime 10.5
7. AlphaFold2

For manuscripts utilizing custom algorithms or software that are central to the research but not yet described in published literature, software must be made available to editors and reviewers. We strongly encourage code deposition in a community repository (e.g. GitHub). See the Nature Portfolio guidelines for submitting code & software for further information.

Data

Policy information about availability of data

All manuscripts must include a data availability statement. This statement should provide the following information, where applicable:

- Accession codes, unique identifiers, or web links for publicly available datasets
- A description of any restrictions on data availability
- For clinical datasets or third party data, please ensure that the statement adheres to our policy

The coordinates and Cryo-EM map of the Torpedo nAChR in complex with ScNtx are deposited in the PDB (7Z14) and EMDB (EMD-14440), respectively. PDB accession codes for structures referenced in this manuscript are: 6UWZ -Torpedo nAChR in complex with alpha-Bgtx and 7KOO - alpha7 nAChR in complex with alpha-Bgtx. Uniprot accession codes for protein sequences referenced in this manuscript are: human muscle nAChR subunit alpha P02708, beta P11230, delta Q07001, epsilon Q04844 and gamma P07510. Uniprot accession codes for short-chain alpha-neurotoxins: P01426, K9MCH1, P80548, P80958, P86095, P01434, P01418, P25675, P86420, P01424, P62388, Q45Z11, P01416, C1IC47, P60775, P60770.

Field-specific reporting

Please select the one below that is the best fit for your research. If you are not sure, read the appropriate sections before making your selection.

☒ Life sciences ☐ Behavioural & social sciences ☐ Ecological, evolutionary & environmental sciences

For a reference copy of the document with all sections, see nature.com/documents/nr-reporting-summary-flat.pdf

Life sciences study design

All studies must disclose on these points even when the disclosure is negative.

Sample size	No statistical tests were used to predetermine sample sizes. Sample sizes for biophysical (MST Nanotemper) and electrophysiology experiments were chosen based on established practice/previous studies and were sufficient as the data were highly reproducible. Nanotemper data were collected in triplicate. All electrophysiology experiments were conducted with a minimum of n=3 and maximum of n=8.<br>For cryo-EM from 1,015,654 particles 26,581 were used for particle reconstruction.
Data exclusions	In the electrophysiology experiments all data are included, unless the recordings did not yield measureable currents or the oocytes lost their voltage clamp during the run.
Replication	All electrophysiological and biophysical experiments were repeated (minimal n=3, maximum n=8) and results were consistent. Results are reported as an average with a standard error of the mean (SEM) or standard deviation (SD). Error bars and dot plots are shown where appropriate.
Randomization	The electrophysiology experiments were conducted in different cells and repeated in different batches. Randomization was not applicable to our study since no predetermined control and sample groups were used.
Blinding	- The electrophysiology experiments at HiQscreen were conducted using an internal reference number and fully blinded to the technician.<br>- All other labs did not use blinding as it would results in untraceable results in experiments.

Reporting for specific materials, systems and methods

We require information from authors about some types of materials, experimental systems and methods used in many studies. Here, indicate whether each material, system or method listed is relevant to your study. If you are not sure if a list item applies to your research, read the appropriate section before selecting a response.

Materials & experimental systems

n/a	Involved in the study	
☐	☒	Antibodies
☒	☐	Eukaryotic cell lines
☒	☐	Palaeontology and archaeology
☒	☐	Animals and other organisms
☒	☐	Human research participants
☒	☐	Clinical data
☒	☐	Dual use research of concern

Methods

n/a	Involved in the study	
☒	☐	ChIP-seq
☒	☐	Flow cytometry
☒	☐	MRI-based neuroimaging

Antibodies

Antibodies used	Megabody Mbc7HopQ was obtained from the Steyaert lab (Uchański et al. Nat. Methods, 2021).
Validation	Megabody Mbc7HopQ was validated and previously published (Uchański et al. Nat. Methods, 2021).
